# Tumor-specific delivery of biologics by a novel T-cell line HOZOT

**DOI:** 10.1038/srep38060

**Published:** 2016-11-30

**Authors:** Teppei Onishi, Hiroshi Tazawa, Yuuri Hashimoto, Makoto Takeuchi, Takeshi Otani, Shuji Nakamura, Fuminori Sakurai, Hiroyuki Mizuguchi, Hiroyuki Kishimoto, Yuzo Umeda, Yasuhiro Shirakawa, Yasuo Urata, Shunsuke Kagawa, Toshiyoshi Fujiwara

**Affiliations:** 1 Department of Gastroenterological Surgery, Okayama University Graduate School of Medicine, Dentistry and Pharmaceutical Sciences, Okayama 700-8558, Japan; 2 Center for Innovative Clinical Medicine, Okayama University Hospital, Okayama 700-8558, Japan; 3 R&D Center, Hayashibara Co., Ltd., Okayama 702-8006, Japan; 4 Laboratory of Biochemistry and Molecular Biology, Graduate School of Pharmaceutical Sciences, Osaka University, Osaka 565-0871, Japan; 5 Oncolys BioPharma, Inc., Tokyo 106-0032, Japan

## Abstract

“Cell-in-cell” denotes an invasive phenotype in which one cell actively internalizes in another. The novel human T-cell line HOZOT, established from human umbilical cord blood, was shown to penetrate a variety of human cancer cells but not normal cells. Oncolytic viruses are emerging as biological therapies for human cancers; however, efficient viral delivery is limited by a lack of tumor-specific homing and presence of pre-existing or therapy-induced neutralizing antibodies. Here, we report a new, intriguing approach using HOZOT cells to transmit biologics such as oncolytic viruses into human cancer cells by cell-in-cell invasion. HOZOT cells were successfully loaded via human CD46 antigen with an attenuated adenovirus containing the fiber protein of adenovirus serotype 35 (OBP-401/F35), in which the telomerase promoter regulates viral replication. OBP-401/F35–loaded HOZOT cells were efficiently internalized into human cancer cells and exhibited tumor-specific killing by release of viruses, even in the presence of anti-viral neutralizing antibodies. Moreover, intraperitoneal administration of HOZOT cells loaded with OBP-401/F35 significantly suppressed peritoneally disseminated tumor growth in mice. This unique cell-in-cell property provides a platform for selective delivery of biologics into human cancer cells, which has important implications for the treatment of human cancers.

“Cell-in-cell” phenomena, in which a whole cell is found in the cytoplasm of another cell, have been reported for decades, although their physiologic significance remains unclear^1^. The formation of cell-in-cell structures occurs following cell-cell contact and commonly results from the engulfment of apoptotic cells by phagocytosis; however, many other types of cell-in-cell structures have been described, such as the invasion of one cell by another^2^. Immune effector cells and their target cells are known to interact in this fashion. Infiltration of immune cells into tumors facilitates direct cell-cell contact and the formation of heterotopic cell-in-cell structures and results in entotic or apoptotic death of the internalized immune cells. This process reflects one of the mechanisms tumor cells employ to evade antitumor immunosurveillance^3^. However, it was recently reported that penetration of tumor cells by immune cells also represents a special form of immune attack, resulting in target cell death as well as self-destruction of the invading immune cells^4^,^5^.

HOZOT is a novel multifunctional regulatory T-cell (Treg) line established from human umbilical cord blood mononuclear cells by co-cultivation with mouse stromal cells. HOZOT cells are characterized by a unique FOXP3/CD4/CD8/CD25-positive phenotype distinct from conventional Tregs, such as FOXP3^+^ natural Tregs or induced Tregs^6^. HOZOT cells exhibit suppressor/helper/cytotoxic activities, and their cytokine expression pattern as determined by mRNA profiling resembles those of Th1 and Th2 T cells, CD8^+^ cytotoxic T lymphocytes, natural killer cells, and Tregs^7^,^8^,^9^. HOZOT cells exhibit cytotoxic activity against various human cancer cell lines but not normal human cells. Furthermore, HOZOT cells can actively penetrate target cancer cells and form cell-in-cell structures, which may be one of the mechanisms by which HOZOT cells exert cytotoxicity against cancer cells^5^. This unique cell-in-cell invasive property led us to examine the potential of using HOZOT cells as tumor-tropic carriers of biologics such as oncolytic viruses.

Oncolytic viruses that can selectively replicate in and lyse infected tumor cells are an emerging therapeutic modality for treating human cancers^10^. These vectors are designed to induce virus-mediated lysis of tumor cells after selective viral propagation within the tumor cell. We developed an attenuated adenovirus, designated OBP-301 (Telomelysin), that drives the expression of *E1* genes under the human telomerase reverse transcriptase (hTERT) promoter^11^, and we confirmed its safety after intratumoral delivery in a phase I clinical trial involving various types of solid tumors^12^. We further modified OBP-301 to express the green fluorescent protein (*GFP*) gene for monitoring viral replication. The resultant adenovirus, designated OBP-401 (TelomeScan), efficiently labeled tumor cells with green fluorescence, enabling us to track viral spread *in vivo*^13^,^14^. Preclinical as well as clinical studies have demonstrated successful virus spread, including spread to the regional lymphatic areas, and promising antitumor effects were shown with intratumoral administration of the virus^15^,^16^. However, systemic intravenous viral delivery was hampered by the presence of pre-existing or therapy-induced neutralizing antibodies^17^.

Here, we show that HOZOT cells loaded with an adenovirus serotype 35 fiber–modified attenuated adenovirus expressing the *GFP* gene (OBP-401/F35) are internalized in human cancer cells as a stealth carrier. These cells form cell-in-cell structures and efficiently destroy the target cells by releasing cytotoxic viruses. Although a variety of cells, including stem and progenitor cells, immune cells, and cancer cells themselves, have been investigated as delivery vehicles for various oncolytic viruses^18^,^19^,^20^,^21^,^22^, the tumor-specific homing and cell-in-cell invasion capabilities of HOZOT cells suggest that they are ideal virotherapeutic cellular carriers.

## Results

### Adenovirus serotype 35 fiber–modified adenovirus is a suitable vector for imparting HOZOT cells with cell-in-cell invasive activity

**Figure 1:**
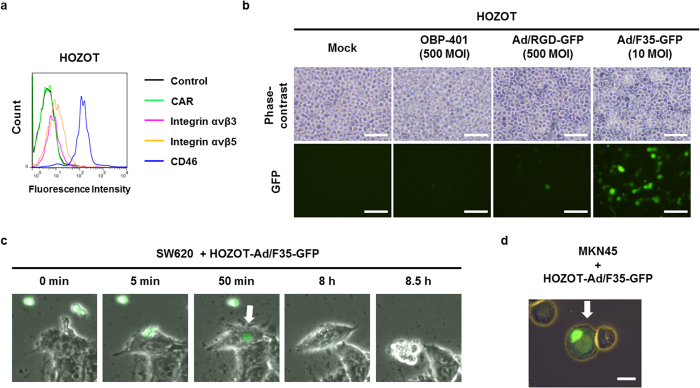
Cell-in-cell activity of HOZOT cells loaded with non-replicative GFP-expressing adenoviruses by adenovirus serotype 35 fiber modification. (**a**) Flow cytometric analysis of adenovirus-related receptor protein (CAR, integrins αVβ3 and αVβ5, and CD46) in HOZOT cells. (**b**) Phase-contrast and fluorescence images of HOZOT cells infected with mock, OBP-401, RGD peptide fiber–modified Ad/RGD-GFP, or adenovirus serotype 35 fiber–modified Ad/F35-GFP. Scale bars: 50 μm. (**c**) Time-lapse images of SW620 cells treated with virus-loaded HOZOT-Ad/F35-GFP. (**d**) Images of MKN45 cells, showing penetration by virus-loaded HOZOT-Ad/F35-GFP cells. White arrows indicate virus-loaded, GFP-positive HOZOT cells. Scale bar: 20 μm.

To identify the most suitable adenovirus vector for loading into HOZOT cells, we first analyzed the expression levels of several adenovirus receptor proteins, such as Coxsackie virus and adenovirus receptor (CAR), integrins αvβ3 and αvβ5, and CD46, on the surface of HOZOT cells using flow cytometric analysis (Fig. 1a). HOZOT cells showed remarkably high CD46 protein expression and slight expression of integrin proteins, but no CAR protein expression was detected, suggesting that HOZOT cells are susceptible to infection with adenovirus serotype 35 fiber–modified adenovirus (Ad/F35), which can bind to CD46 protein. To assess whether the adenovirus infection efficiency depends on cell surface expression of adenovirus receptor protein, we analyzed the efficacy of GFP induction in HOZOT cells infected with various types of fiber-modified adenovirus vectors that express the GFP protein (Fig. 1b). Consistent with receptor protein expression level, non-replicative Ad/F35-GFP, which binds to CD46, induced GFP expression more efficiently in HOZOT cells than either non-replicative RGD peptide fiber-modified adenovirus (Ad/RGD), which binds to integrins αvβ3 and αvβ5, or conditionally tumor-specific replicative adenovirus (OBP-401), which binds to CAR. These results indicate that the F35 fiber–modified adenovirus is the most suitable vector for loading into HOZOT cells through binding to CD46.

To investigate whether virus infection affects the cell-in-cell activity of HOZOT cells, SW620 human colorectal cancer cells and MKN45 human gastric cancer cells were exposed to virus-loaded HOZOT-Ad/F35 cells pre-infected with Ad/F35-GFP (Fig. 1c,d). As shown in Fig. 1c, virus-loaded HOZOT-Ad/F35-GFP cells penetrated and formed cell-in-cell structures in SW620 cells as early as 50 min after treatment. Virus-loaded HOZOT-Ad/F35-GFP cells also penetrated and formed cell-in-cell structures in MKN45 cells (Fig. 1d). These results suggest that virus-loaded HOZOT cells maintain cell-in-cell activity identical to virus-free HOZOT cells.

### HOZOT cell–based delivery of OBP-401/F35 induces tumor-specific cell death

**Figure 2:**
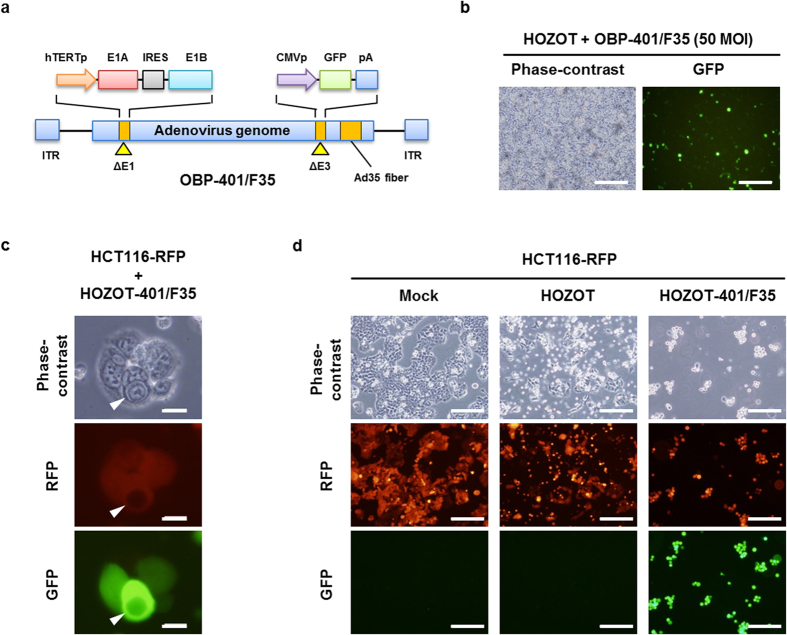
HOZOT cell–based delivery of OBP-401/F35 into human tumor cells. (**a**) Schematic diagram of the OBP-401/F35 structure. OBP-401/F35 is a telomerase-specific, replication-competent, adenovirus serotype 35 fiber–modified adenovirus serotype 5, in which the *hTERT* promoter drives the expression of the IRES-linked *E1A* and *E1B* genes and the cytomegalovirus (CMV)promoter drives the expression of the *GFP* gene inserted in the E3 region. (**b**) Phase-contrast and fluorescence images of HOZOT cells infected with OBP-401/F35. Scale bar: 100 μm. (**c**) Phase-contrast and fluorescence images of HCT116-RFP cells after treatment with virus-loaded HOZOT-401/F35 cells. White arrowheads indicate HOZOT cells that penetrated HCT116-RFP cells. Scale bars: 20 μm. (**d**) Phase-contrast and fluorescence images of HCT116-RFP cells after treatment with mock, virus-free HOZOT cells, or virus-loaded HOZOT-401/F35 cells. Scale bars: 200 μm.

To evaluate the potential for using HOZOT cells as carriers for oncolytic adenoviruses, we generated a telomerase-specific, replication-competent oncolytic adenovirus, OBP-401/F35, in which the fiber was modified to adenovirus serotype 35 (Fig. 2a). Adenovirus serotype 35 recognizes human CD46 and, moreover, CD46 expression on the surface of malignant tumor cells is up-regulated^23^,^24^. Indeed, OBP-401/F35 efficiently reduced the viability of all human cancer cells examined (SW620, MKN45, HCT116) that expressed both CAR and CD46 proteins (Suppl. Fig. 1). When HOZOT cells were infected with OBP-401/F35, GFP expression was efficiently induced in HOZOT cells (Fig. 2b). In contrast, to assess whether OBP-401/F35 replicates in HOZOT cells, we analyzed the expression of *hTERT* mRNA and the copy number of adenoviral E1A in HOZOT cells. HOZOT cells showed lower *hTERT* mRNA expression compared to cancer cells and low-level virus replication until 48 hours after infection (Suppl. Fig. 2). When red fluorescent protein (RFP)-expressing HCT116 human colon cancer cells (HCT116-RFP cells) were treated with OBP-401/F35–loaded HOZOT cells (HOZOT-401/F35 cells), the HOZOT-401/F35 cells penetrated and induced GFP expression in HCT116-RFP cells (Fig. 2c,d). These results indicate that virus-loaded HOZOT-401/F35 cells have a great potential for use in the delivery of telomerase-specific replicating oncolytic adenoviruses into tumor cells.

**Figure 3:**
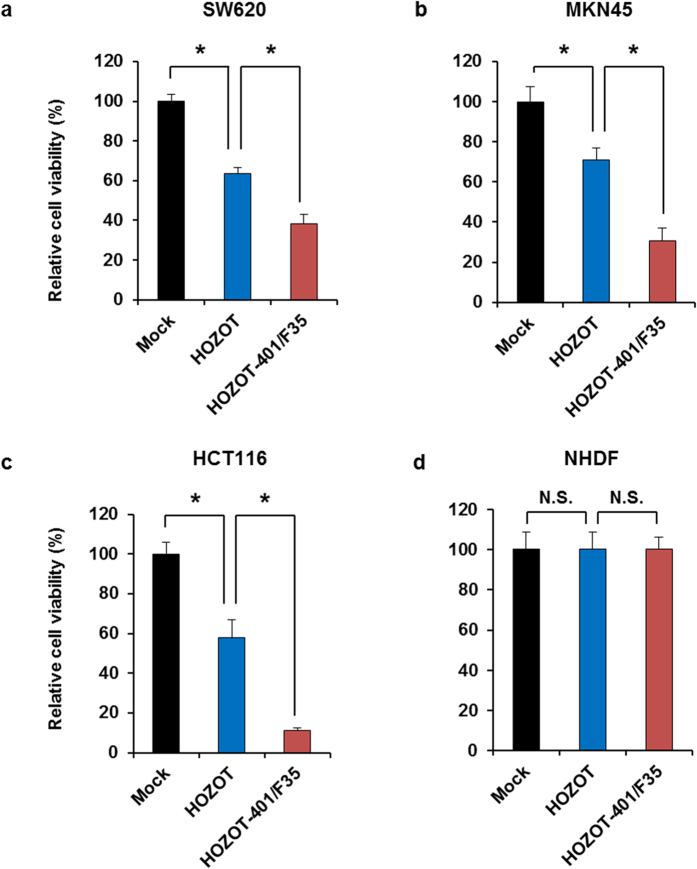
Tumor-specific cytopathic effect of oncolytic adenovirus–loaded HOZOT-401/F35 cells. Human gastrointestinal cancer cells, SW620 (**a**), MKN45 (**b**), HCT116 (**c**), and NHDFs (**d**) were treated with mock, virus-free HOZOT cells, or virus-loaded HOZOT-401/F35 cells. Cell viability was calculated relative to that of the mock-treated group, which was set at 100%. Cell viability data are expressed as mean values ± SD (n = 5). *P < 0.05. N.S., not significant.

To assess whether virus-loaded HOZOT-401/F35 cells induce tumor-specific virus-mediated cytopathic effects, we compared the *in vitro* antitumor activity of virus-loaded HOZOT-401/F35 cells and virus-free HOZOT cells against several human cancer cell lines (SW620, MKN45, and HCT116) and normal human fibroblasts (NHDFs). Virus-free HOZOT cells significantly reduced the viability of all human cancer cells examined in a dose-dependent manner (Suppl. Fig. 3). Compared with virus-free HOZOT cells, virus-loaded HOZOT-401/F35 cells induced a greater cytopathic effect in all human cancer cell lines examined (Fig. 3). In contrast, both virus-free and virus-loaded HOZOT-401/F35 cells showed no cytotoxic effect against NHDFs (Fig. 3 and Suppl. Fig. 3). These results suggest that virus-loaded HOZOT-401/F35 cells possess greater cytopathic activity than virus-free HOZOT cells as a result of their ability to deliver oncolytic adenoviruses to target cells.

### OBP-401/F35 loaded into HOZOT cells overcomes antibody-mediated virus neutralization

**Figure 4:**
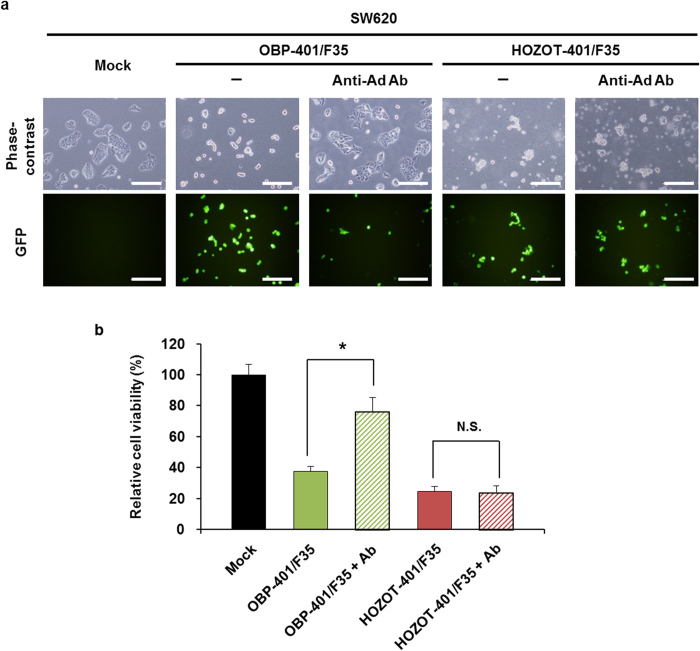
HOZOT cell–mediated protection of OBP-401/F35 from antibody-mediated virus neutralization. (**a**) Phase-contrast and fluorescence images of SW620 cells treated with mock, OBP-401/F35, or virus-loaded HOZOT-401/F35 cells in the presence or absence of neutralizing antibody. Scale bars: 200 μm. (**b**) SW620 cells treated with mock, OBP-401/F35, or virus-loaded HOZOT-401/F35 cells. Cell viability was calculated relative to that of the mock-treated group, which was set at 100%. Cell viability data are expressed as mean values ± SD (n = 5). *P < 0.05. N.S., not significant.

Carrier cells have shown promise as vehicles for the systemic administration of oncolytic viruses^25^,^26^, which is often hampered by antibody-mediated virus neutralization. We therefore investigated whether virus-loaded HOZOT-401/F35 cells exhibit antitumor effects in the presence of neutralizing antibody. In the absence of neutralizing antibody, both virus-loaded HOZOT-401/F35 cells and naked OBP-401/F35 efficiently induced GFP expression in SW620 human colon cancer cells (Fig. 4a). In contrast, anti-adenovirus neutralizing antibody inhibited the virus-mediated induction of GFP expression by naked OBP-401/F35, but the antibody did not inhibit virus-loaded HOZOT-401/F35 cell–mediated induction of GFP expression. Moreover, the neutralizing antibody significantly attenuated the virus-mediated antitumor effect of naked OBP-401/F35 but not the cell-mediated antitumor effect of virus-loaded HOZOT-401/F35 cells (Fig. 4b). These results indicate that virus-loaded HOZOT-401/F35 cells are highly resistant to antibody-mediated virus neutralization.

### Virus-loaded HOZOT-401/F35 cells suppress the formation of tumor spheres through virus delivery

**Figure 5:**
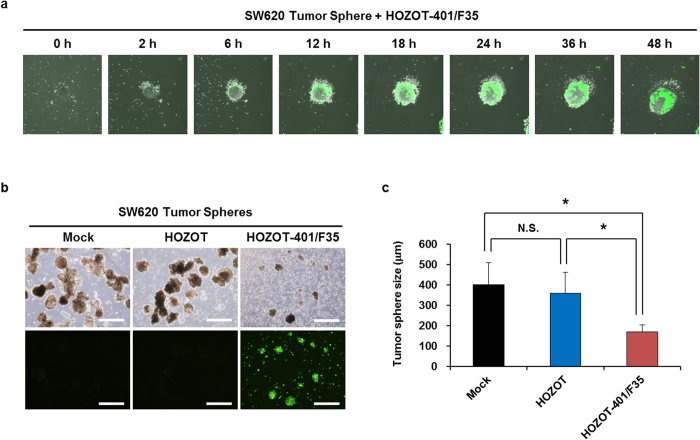
Suppression of SW620 tumor sphere formation by treatment with virus-loaded HOZOT-401/F35 cells. (**a**) Time-lapse images of tumor spheres treated with virus-loaded HOZOT-401/F35 cells. (**b**) Phase-contrast and fluorescence images of tumor spheres from SW620 cells after treatment with mock, virus-free HOZOT cells, or virus-loaded HOZOT-401/F35 cells (left). Scale bars: 500 μm. Tumor size (diameter) was calculated for each group of tumor spheres treated with mock (n = 29), virus-free HOZOT cells (n = 30), or virus-loaded HOZOT-401/F35 cells (n = 18) (right). Data are expressed as mean values ± SD. *P < 0.05. N.S., not significant.

To further confirm the therapeutic potential of virus-loaded HOZOT-401/F35 cells, we investigated the treatment dynamics of SW620 cell–derived tumor spheres after treatment with virus-loaded HOZOT-401/F35 cells. Time-lapse imaging spanning 48 hours after treatment showed that virus-loaded HOZOT-401/F35 cells induced GFP expression on the periphery of the tumor sphere (Fig. 5a and Suppl. Video 1). Moreover, time-lapse imaging spanning 5 days after treatment showed that HOZOT-401/F35 cells induced GFP expression and subsequent cell death in tumor spheres composed of SW620 cells (Supple. Video 2). In addition, virus-loaded HOZOT-401/F35 cells significantly suppressed the size of SW620 tumor spheres, although virus-free HOZOT cells did not affect the formation of tumor spheres (Fig. 5b). These results suggest that virus-loaded HOZOT-401/F35 cells efficiently deliver OBP-401/F35 virus to sphere-forming tumor cells, resulting in the suppression of tumor sphere formation.

### *In vivo* inhibition of tumor dissemination by intraperitoneal injection of virus-loaded HOZOT-401/F35 cells

**Figure 6:**
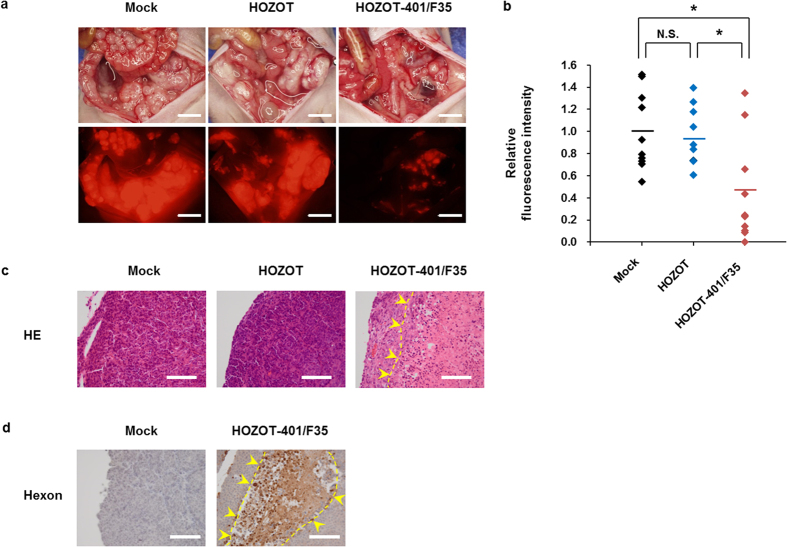
Suppression of the growth of peritoneally disseminated tumors derived from HCT116-RFP cells by treatment with virus-loaded HOZOT-401/F35 cells. (**a**) Macroscopic appearance and fluorescence images of the abdominal cavity at 14 days after treatment with mock, virus-free HOZOT cells, and virus-loaded HOZOT-401/F35 cells. Scale bars: 5 mm. (**b**) Relative fluorescence intensity of peritoneally disseminated tumors in mice treated with mock, virus-free HOZOT cells, and virus-loaded HOZOT-401/F35 cells. Data are expressed as mean values ± SD (n = 10). Statistical significance was determined using the Student’s *t* test. *P < 0.05. N.S., not significant. (**c**) Histopathologic examination of excised tumors stained with hematoxylin and eosin. Large necrotic area is shown with yellow arrowheads. Scale bars: 100 μm. (**d**) Immunohistologic analysis of adenoviral hexon protein in mock-treated and virus-loaded HOZOT-401/F35–treated tumor tissues. Positive staining is reddish brown (yellow arrowheads). Scale bars: 100 μm.

Peritoneal dissemination of cancer cells is one of the main causes of cancer mortality. For preclinical evaluation of the therapeutic potential of virus-loaded HOZOT cells, we investigated whether intraperitoneal injection of virus-loaded HOZOT-401/F35 cells prevents peritoneal metastasis of HCT116-RFP human colorectal cancer cells *in vivo*. Virus-loaded HOZOT-401/F35 cells or virus-free HOZOT cells were injected intraperitoneally in mice 1 week after injection of HCT116-RFP cells into the peritoneal cavity. At 2 weeks after treatment, marked peritoneal dissemination was observed in mice treated with mock control or virus-free HOZOT cells, whereas peritoneal dissemination was reduced in mice treated with virus-loaded HOZOT-401/F35 cells (Fig. 6a). Quantitative analysis of RFP intensity in the peritoneal cavity revealed that virus-loaded HOZOT-401/F35 cells significantly inhibited the peritoneal dissemination of HCT116-RFP cells when compared with mock control or virus-free HOZOT cells (Fig. 6b). Histopathologic analysis revealed large necrotic areas in tumor tissues treated with virus-loaded HOZOT-401/F35 cells but not in tissues treated with mock control or virus-free HOZOT cells (Fig. 6c). The expression of virus-related hexon protein was observed in tumors treated with virus-loaded HOZOT-401/F35 cells but not in mock control–treated tumors (Fig. 6d). Treatment with virus-loaded HOZOT-401/F35 cells significantly increased the median survival time (MST) of HCT116-RFP disseminated tumor–bearing mice when compared to mice treated with mock control or virus-free HOZOT cells (46.5 day vs. 38 days; *P* < 0.05). as determined by the Kaplan-Meier method. In contrast, there was no significant difference in the MST between mock control– and virus-free HOZOT cell–treated mice exhibiting peritoneal dissemination (38 days vs. 25.5 days). These results suggest that virus-loaded HOZOT-401/F35 cells efficiently inhibit peritoneal dissemination of tumors through delivery of oncolytic adenoviruses.

## Discussion

The formation of cell-in-cell structures is a biological process following cell-cell contact in inflammatory and tumor tissues^1^,^2^. Although the biological properties of cell-in-cell structures remain unclear, the cell-in-cell activity by which immune cells penetrate the cytoplasm of tumor cells has been shown to result in programmed cell-in-cell death^4^,^5^. HOZOT cells are a novel cord blood–derived line of Tregs that exhibit multiple properties, such as cytotoxic activity and cell-in-cell activity against human tumor cells^5^. These findings have led to considerable recent research exploring the therapeutic effects of combining HOZOT cells and oncolytic viruses. Here, we investigated the potential of using HOZOT cells as carriers for efficient transmission of the oncolytic adenovirus OBP-401/F35 into human cancer cells. Virus-loaded HOZOT-401/F35 cells significantly reduced the viability of human cancer cells, but not that of normal cells, through virus delivery. In the presence of anti-adenovirus neutralizing antibody, virus-loaded HOZOT-401/F35 cells, but not naked OBP-401/F35 virus, efficiently reduced the viability of human cancer cells. Moreover, both *in vitro* sphere formation and *in vivo* peritoneal dissemination of tumor cells were significantly suppressed in mice treated with virus-loaded HOZOT-401/F35 cells, resulting in a significant increase in the survival rate of tumor-bearing mice. Thus, this unique tumor-specific cell-in-cell property of HOZOT cells confirms their potential for use as a delivery system to target disseminated metastatic tumor cells in oncolytic virotherapy.

Carrier cell–based delivery of oncolytic viruses has the advantage of direct virus delivery into disseminated metastatic tumor cells by systemic administration^25^,^26^. Normal mesenchymal and neural stem cells have been investigated as carrier cells for oncolytic viruses because of their tumor tropism induced by tumor-derived chemoattractants^26^. However, stem cell–based delivery systems lack the cell-in-cell invasion property, and the tumor-homing ability of stem cells would be limited to the stromal areas surrounding tumor cells. Although a variety of immune cells, including cytokine-induced killer cells, dendritic cells, and myeloid-derived suppressor cells, can take up viruses, no mechanism to then transmit the viruses to tumor cells has been elucidated^20^,^27^,^28^. In the present study, we examined the novel HOZOT line of human T cells, which exhibit cell-in-cell invasion and subsequent cytotoxic effects against tumor cells. Virus-loaded HOZOT-Ad/F35-GFP cells and HOZOT-401/F35 cells maintained the cell-in-cell activity in target tumor cells. Virus-loaded HOZOT-401/F35 cells showed a greater antitumor effect than virus-free HOZOT cells, suggesting that the antitumor effect is virus mediated. In addition, virus-loaded HOZOT-401/F35 cells exhibited no cytotoxic effect against normal fibroblasts or virus-free HOZOT cells. Therefore, due to their cell-in-cell activity, HOZOT cells are ideal carriers for direct, tumor-specific intracellular delivery of oncolytic adenoviruses to induce the death of cancer cells.

The presence of pre-existing neutralizing antibodies is a major obstacle to oncolytic adenovirus–mediated antitumor therapies, as most cancer patients have been previously exposed to adenoviruses. Although in a previous study we confirmed the regional spread of intratumorally injected adenovirus vectors to the lymphatic vessels^29^, systemic delivery of oncolytic adenoviruses is hampered by the circulation of anti-adenovirus neutralizing antibodies in the plasma^30^. In the current study, we demonstrated that the penetration of virus-loaded HOZOT-401/F35 cells into tumor cells circumvents antibody-mediated virus neutralization, whereas infection with naked OBP-401/F35 virus is significantly attenuated by neutralizing antibody. We previously showed that transgene expression induced by repeated intratumoral injection of a replication-deficient adenovirus vector can be detected in tumor tissues throughout the treatment period, despite the presence of neutralizing anti-adenovirus antibodies^29^. Therefore, once virus-loaded HOZOT-401/F35 cells have trafficked to the tumor site, a plentiful supply of viruses would be available due to lysis of targeted tumor cells, thus providing a prolonged antitumor effect. Our data indicate that HOZOT cells are ideal stealth-type carrier cells for systemic delivery of oncolytic adenoviruses due to their cell-in-cell activity.

Peritoneal dissemination is one of the most frequent causes of mortality in cancer patients^31^. Although multimodal approaches such as intraperitoneal chemotherapy and hyperthermic intraperitoneal chemoperfusion (HIPEC) have been explored^32^, there is no standard treatment to effectively prevent peritoneal dissemination. We recently reported that intraperitoneal administration of OBP-301 in combination with cisplatin efficiently suppresses the peritoneal dissemination of ovarian cancer cells *in vivo*^33^. Intraperitoneal administration of OBP-401 is also useful for *in vivo* identification of peritoneally disseminated human cancer cells in fluorescence-guided surgery^34^. However, local immunologic responses involving peritoneal macrophages, in addition to antibody-mediated virus neutralization, may hamper the antitumor effects of this strategy. In the current study, however, virus-loaded HOZOT-401/F35 cells significantly suppressed the *in vitro* formation of tumor spheres that mimic disseminated tumor nodules. Moreover, intraperitoneal administration of virus-loaded HOZOT-401/F35 cells significantly inhibited the *in vivo* growth of peritoneally disseminated tumor cells and prolonged the survival of tumor-bearing mice. Although allogeneic HOZOT cells may induce the immune response in cancer patients, autologous HOZOT cells from cancer patients may be available in the future because our collaborators have recently established HOZOT-like cells, which show the similar cell-in-cell activity, from peripheral T lymphoid cells (personal communication). Thus, virus-loaded HOZOT-401/F35 cells represent a more promising antitumor reagent than naked oncolytic adenoviruses for the treatment of peritoneal dissemination in cancer patients.

Recent successes in the development of cancer immunotherapies have underscored the high capacity of the immune system to eradicate tumors, generating renewed enthusiasm for identifying methods to enhance antitumor immune responses in cancer patients. Many researchers are looking for attractive candidate drugs that can be combined with immunotherapy agents to activate host immunity^35^. We previously reported that oncolysis induced by telomerase-specific oncolytic adenovirus infection might be the most effective stimulus for immature dendritic cells to induce specific activity against human cancer cells (i.e., immunogenic cancer cell death)^36^,^37^. As HOZOT cells are cytotoxic regulatory T cells, virus-loaded HOZOT-401/F35 cells may be effective both as a direct cytotoxic agent and an immunostimulatory mediator that induces specific cytotoxic T lymphocytes against the remaining antigen-bearing tumor cells. Telomerase-specific oncolytic adenoviruses might also alter the tumor microenvironment by stimulating host immune cells to produce endogenous cytokines such as INF-γ^38^. Thus, a possible strategy for using virus-loaded HOZOT-401/F35 cells includes combination with immunotherapy.

In conclusion, we clearly demonstrated that virus-loaded HOZOT cells possess great potential for tumor-specific intracellular delivery of oncolytic adenoviruses through their cell-in-cell invasion activity. The ability of virus-loaded HOZOT cells to overcome antibody-mediated virus neutralization and improve virus delivery to multiple disseminated tumors makes them promising antitumor agents. The unique cell-in-cell property of virus-loaded HOZOT cells provides a platform for selective delivery of biologics into human cancer cells, an outcome that has important implications for the treatment of human cancers.

## Materials and Methods

### Cell lines

SW620 and HCT116 human colorectal cancer cells were obtained from the American Type Culture Collection (Manassas, VA, USA). HCT116-RFP human colorectal cancer cells that express RFP were purchased from AntiCancer, Inc. (San Diego, CA, USA). MKN45 human gastric cancer cells were obtained from the Japanese Collection Research Bioresources (JCRB, Osaka, Japan). NHDF cells were purchased from Kurabo (Osaka, Japan). SW620, HCT116-RFP, MKN45, and NHDF cells were maintained in RPMI-1640 medium supplemented with 10% fetal bovine serum, 100 U/mL penicillin, and 100 mg/mL streptomycin. Cells were routinely maintained at 37 °C in a humidified atmosphere containing 5% CO_2_.

Novel human HOZOT T cells were obtained from Hayashibara Biochemical Laboratories, Inc. (Okayama, Japan). HOZOT cells were previously established from human umbilical cord blood^6^ and characterized as a cytotoxic Treg line with cell-in-cell activity^5^. HOZOT cells were maintained over mouse normal stromal ST2 cells (purchased from the RIKEN BioResource Center [Ibaraki, Japan]) and cultured in RPMI-1640 medium supplemented with 10% fetal bovine serum, 100 U/mL penicillin, and 100 mg/mL streptomycin. Prior to experiments, HOZOT cells were purified by Ficoll-Paque to deplete debris derived from dead mouse stromal cells.

### Recombinant adenoviruses and neutralizing antibody

The study utilized the previously constructed and characterized telomerase-specific, conditionally replicating, GFP-expressing adenovirus OBP-401 (TelomeScan), in which the promoter element of the human telomerase reverse transcriptase (*hTERT*) gene drives the expression of the internal ribosome entry site (IRES)-linked *E1A* and *E1B* genes. For loading into CAR-negative HOZOT cells, the fiber knob of OBP-401 was modified from adenovirus serotype 5 (which binds CAR) to adenovirus serotype 35 (which binds CD46) or RGD-peptide fiber (which binds integrins αvβ3 and αvβ5). The resulting E1A-deletant, non-replicative, GFP-expressing adenoviruses with RGD peptide–modified fibers (Ad/RGD-GFP) and adenovirus serotype 35–modified fibers (Ad/F35-GFP) were used to evaluate virus infection of HOZOT cells. Recombinant viruses were purified by ultracentrifugation using a cesium chloride step gradient, and virus titer was determined by plaque-forming assay using 293 cells. Viruses were stored at −80 °C. To attenuate the effect of recombinant adenovirus, sera containing neutralizing antibody against adenovirus serotype 5 were purchased from Denka Seiken, Co., Ltd., (Tokyo, Japan).

### Flow cytometric analysis

HOZOT cells were labeled with mouse monoclonal anti-CAR (RmcB; Upstate Biotechnology, Lake Placid, NY, USA), anti-human integrin αvβ3 (LM609; Millipore, Temecula, CA, USA), anti-human integrin αvβ5 (P1F6; Millipore), or anti-human CD46 (E4.3; BD Pharmingen, San Diego, CA, USA) antibody for 30 minutes at 4 °C. Cells were then incubated with fluorescein isothiocyanate (FITC)-conjugated rabbit anti-mouse IgG secondary antibody (Zymed Laboratories, Inc., South San Francisco, CA, USA) and analyzed by flow cytometry (FACS Array; BD Biosciences, Franklin Lakes, NJ, USA).

### Fluorescent microscopy

HOZOT cells were treated with mock control, OBP-401 (500 multiplicity if infection [MOI]), Ad/RGD-GFP (500 MOI), Ad/F35-GFP (10 MOI), or OBP-401/F35 (50 MOI). Expression of GFP was assessed and photographed using an IX71 fluorescent microscope (Olympus, Tokyo, Japan).

### Time-lapse confocal laser microscopy

Virus-loaded HOZOT-401/F35 cells were obtained by infecting HOZOT cells with OBP-401/F35 (5 MOI) for 24 hours and washing twice with fresh medium to remove the non-internalized adenovirus. SW620 cells, MKN45 cells, and SW620 tumor spheres were then treated with virus-loaded HOZOT/401-F35. The cell-in-cell activity of HOZOT cells within tumor cells was evaluated using time-lapse confocal laser microscopy (FV10i; Olympus).

### Cell viability assay

Cells were seeded in 96-well plates at a density of 1 × 10^3^ cells/well 24 hours before treatment. Cells were then treated with mock control, virus-free HOZOT cells, or virus-loaded HOZOT-401/F35 cells for 48 hours. Cell viability was determined using a Cell Proliferation Kit II (Roche Molecular Biochemicals, Indianapolis, IN, USA).

### Sphere formation assay

SW620 cells were seeded in ultra-low-attachment 6-well plates. At 2 weeks after seeding, tumor sphere–forming SW620 cells were treated with mock control, virus-free HOZOT cells, or virus-loaded HOZOT-401/F35 cells. Tumor sphere size was measured using an IX71 fluorescent microscope.

### *In vivo* peritoneal dissemination model

All animal studies were conducted in accordance with the Policy on the Care and Use of the Laboratory Animals, Okayama University. Animal experimental protocols were approved by the Ethics Review Committee for Animal Experimentation of Okayama University School of Medicine (No. OKU-2011062). HCT116-RFP cells (5 × 10^6^ cells/mouse) were inoculated into the peritoneal cavity of 6-week-old female athymic nude mice (CLEA Japan). One week after inoculation, mice were treated with mock control, virus-free HOZOT cells (2 × 10^7^ cells/mouse), or virus-loaded HOZOT-401/F35 cells (2 × 10^7^ cells/mouse). Two weeks after treatment, red fluorescence photographs were taken under a fluorescent microscope from 10 mice in each group, after laparotomy. Tumor volume was calculated based on red fluorescence intensity using ImageJ software. The survival rate of mice with HCT116-RFP xenograft tumors was assessed until 60 days after inoculation.

### Histopathologic analysis and immunohistochemistry

Disseminated tumors were harvested from mice treated with mock control, virus-free HOZOT cells, or virus-loaded HOZOT-401/F35 cells. Tumors were fixed in 10% neutralized formalin and embedded in paraffin blocks. Sections were stained with hematoxylin/eosin and analyzed under light microscopy. Sections were also prepared for immunohistochemical examination. After deparaffinization and rehydration, antigen retrieval was performed in 10 mM citrate buffer (pH 6.0). Tissue sections were incubated with mouse anti-adenovirus hexon monoclonal antibody (MAB805; Millipore). Immunoreactive signals were visualized using 3,3′-diaminobenzidine tetrahydrochloride solution, and nuclei were counterstained with hematoxylin.

### Statistical analysis

Data are expressed as means ± standard deviation (SD). A one-way ANOVA followed by a Bonferroni multiple-group camparison test was used to compare differences between groups. The log-rank test was also used to compare differences in survival rate between groups of mice. Statistical significance was defined as a *P* value less than 0.05.

## Additional Information

**How to cite this article**: Onishi, T. *et al*. Tumor-specific delivery of biologics by a novel T-cell line HOZOT. *Sci. Rep.* **6**, 38060; doi: 10.1038/srep38060 (2016).

**Publisher's note:** Springer Nature remains neutral with regard to jurisdictional claims in published maps and institutional affiliations.

## Supplementary Material

Supplementary Video 1

Supplementary Video 2

Supplementary Information
