# Supplementary material for: Tumor-specific delivery of biologics by a novel T-cell line HOZOT
Source: Sci Rep. 2016 Nov 30;6:38060. doi: 10.1038/srep38060 (PMC5129011; doi:10.1038/srep38060)
Supplement: Supplementary Information [file srep38060-s3.pdf]

Supplementary Information

Tumor-specific delivery of biologics by a novel T-cell line HOZOT

Teppei Onishi[1], Hiroshi Tazawa[1,2], Yuuri Hashimoto[1], Makoto Takeuchi[3], Takeshi Otani[3], Shuji Nakamura[3], Fuminori Sakurai[4], Hiroyuki Mizuguchi[4], Hiroyuki Kishimoto[1], Yuzo Umeda[1], Yasuhiro Shirakawa[1], Yasuo Urata[5], Shunsuke Kagawa[1] & Toshiyoshi Fujiwara[1]

[1]Department of Gastroenterological Surgery, Okayama University Graduate School of Medicine, Dentistry and Pharmaceutical Sciences, Okayama 700-8558, Japan. [2]Center for Innovative Clinical Medicine, Okayama University Hospital, Okayama 700-8558, Japan. [3]R&D Center, Hayashibara Co., Ltd., Okayama 702-8006, Japan. [4]Laboratory of Biochemistry and Molecular Biology, Graduate School of Pharmaceutical Sciences, Osaka University, Osaka 565-0871, Japan. [5]Oncolys BioPharma, Inc., Tokyo 106-0032, Japan.

Supplementary Materials and Methods

Supplementary Figure 1

Expression of adenovirus receptors and cytopathic effect of OBP-401/F35 in human cancer cells.

Supplementary Figure 2

Expression of *hTERT* mRNA and replication rate of OBP-401/F35 in HOZOT cells.

Supplementary Figure 3

HOZOT cell-mediated cytotoxic effect against human cancer cells and normal fibroblasts.

Supplementary Video 1

Supplementary Video 2

Supplementary Materials and Methods

Cell lines. H1299 and A549 human lung cancer cells and SK-BR-3 human breast cancer cells were obtained from the American Type Culture Collection. T.Tn human esophageal cancer cells and MKN7 human gastric cancer cells were purchased from the Japanese Collection Research Bioresources. H1299, T.Tn, and MKN7 cells were maintained in RPMI-1640 medium, and A549 cells were maintained in Dulbecco's modified Eagle's medium containing a nutrient mixture (Ham's F-12). SK-BR-3 cells were maintained in McCoy's 5A medium. All media were supplemented with 10% heat-inactivated FBS, 100 units/mL penicillin, and 100 mg/mL streptomycin. Cells were maintained at 37°C in a humidified atmosphere with 5% CO_2.

Flow cytometric analysis. Human cancer cells were labeled with mouse monoclonal anti-CAR (RmcB; Upstate Biotechnology) or anti-human CD46 (E4.3; BD Pharmingen) antibody for 30 minutes at 4°C. The cells were then incubated with FITC-conjugated rabbit anti-mouse IgG secondary antibody (Zymed Laboratories) and analyzed by flow cytometry (FACS Array; BD Biosciences).

Cell viability assay. Cells were seeded in 96-well plates at a density of 1×10^3 cells/well 24 hours before infection. Cells were infected with OBP-401/F35 at the indicated MOI or treated with HOZOT cells at the indicated effector-to-target (E/T) ratio. Cell viability was determined using a Cell Proliferation Kit II (Roche).

Quantitative real-time reverse transcription (RT)-PCR analysis. Total RNA was extracted from cells using a miRNeasy Mini Kit (Qiagen). After synthesis of cDNA using 100 ng of total RNA, the levels of *hTERT* and glyceraldehydes-3-phosphate dehydrogenase (*GAPDH*) mRNA expression were determined by quantitative real-time RT-PCR using a StepOnePlusTM real-time PCR system (Applied Biosystems) and TaqMan Gene Expression Assays (Applied Biosystems). The expression of *hTERT* mRNA was defined based on the threshold cycle (Ct), and relative expression levels were calculated using the $2^{-\Delta\Delta Ct}$ method after normalization with reference to the expression of *GAPDH* mRNA.

To examine the *E1A* copy number in OBP-401/F35-infected HOZOT cells, genomic DNA was extracted from serially diluted viral stocks and HOZOT cells infected with OBP-401/F35 at an MOI of 5 or 10 plaque-forming units (PFU)/cell using a QIAmp DNA Mini Kit (Qiagen). *E1A* copy number was also determined using the TaqMan real-time PCR system (Applied Biosystems).

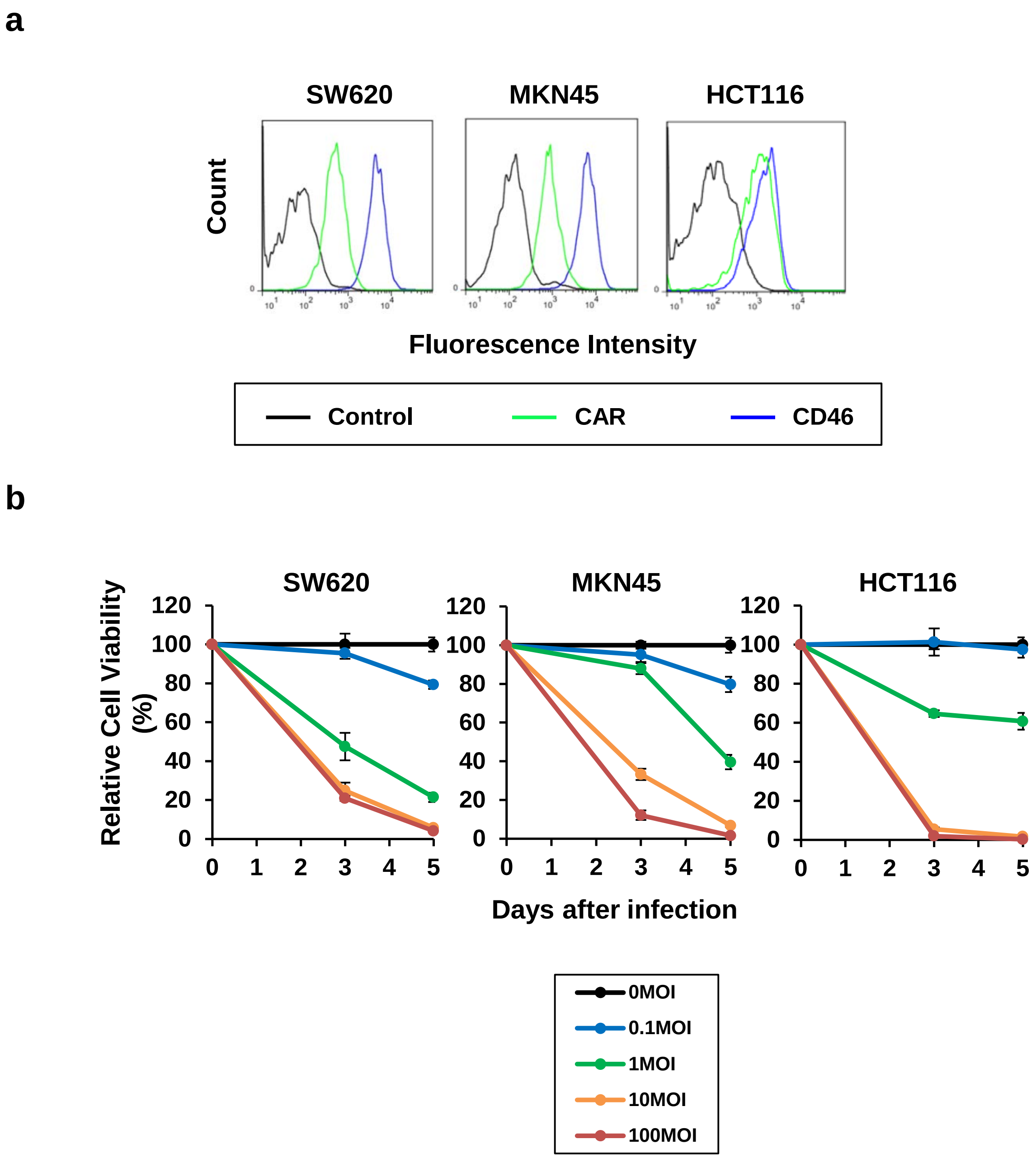

Supplementary Figure 1

Expression of adenovirus receptors and cytopathic effect of OBP-401/F35 in human cancer cells. (**a**) Flow cytometric analysis of CAR and CD46 expression in human cancer cells (SW620, MKN45, HCT116). (**b**) *In vitro* cytopathic effect of OBP-401/F35 in human cancer cells (SW620, MKN45, HCT116). Human cancer cells were infected with OBP-401/F35 at the indicated MOI, and cell viability was quantified over 5 days using the XTT assay. Cell viability was calculated relative to that of the mock-infected group on each day, which was set at 100%. Cell viability data are expressed as mean values ± SD (n = 5).

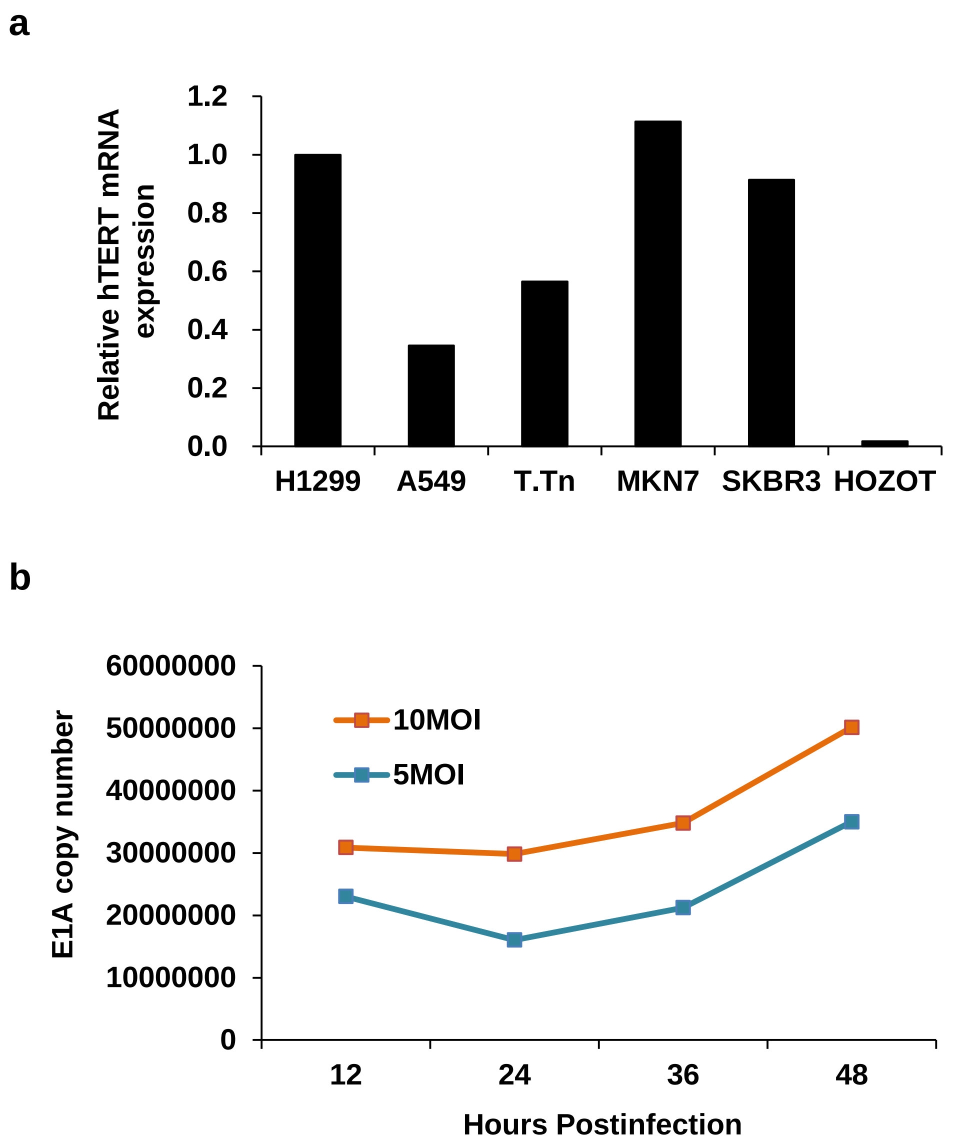

Supplementary Figure 2

Expression of *hTERT* mRNA and replication rate of OBP-401/F35 in HOZOT cells. (**a**) Expression of *hTERT* mRNA in human cancer cells and HOZOT cells as determined using quantitative real-time RT-PCR. The relative level of *hTERT* mRNA was calculated after normalization with reference to the expression of *GAPDH* mRNA. (**b**) Quantitative measurement of viral DNA replication in HOZOT cells infected with OBP-401/F35. HOZOT cells were infected with OBP-401/F35 at an MOI of 5 or 10 PFU/cell, and *E1A* copy number was analyzed over the following 2 days by quantitative real-time RT-PCR. The *E1A* copy number at 2 hours after infection was set at 1, and relative copy numbers were then plotted.

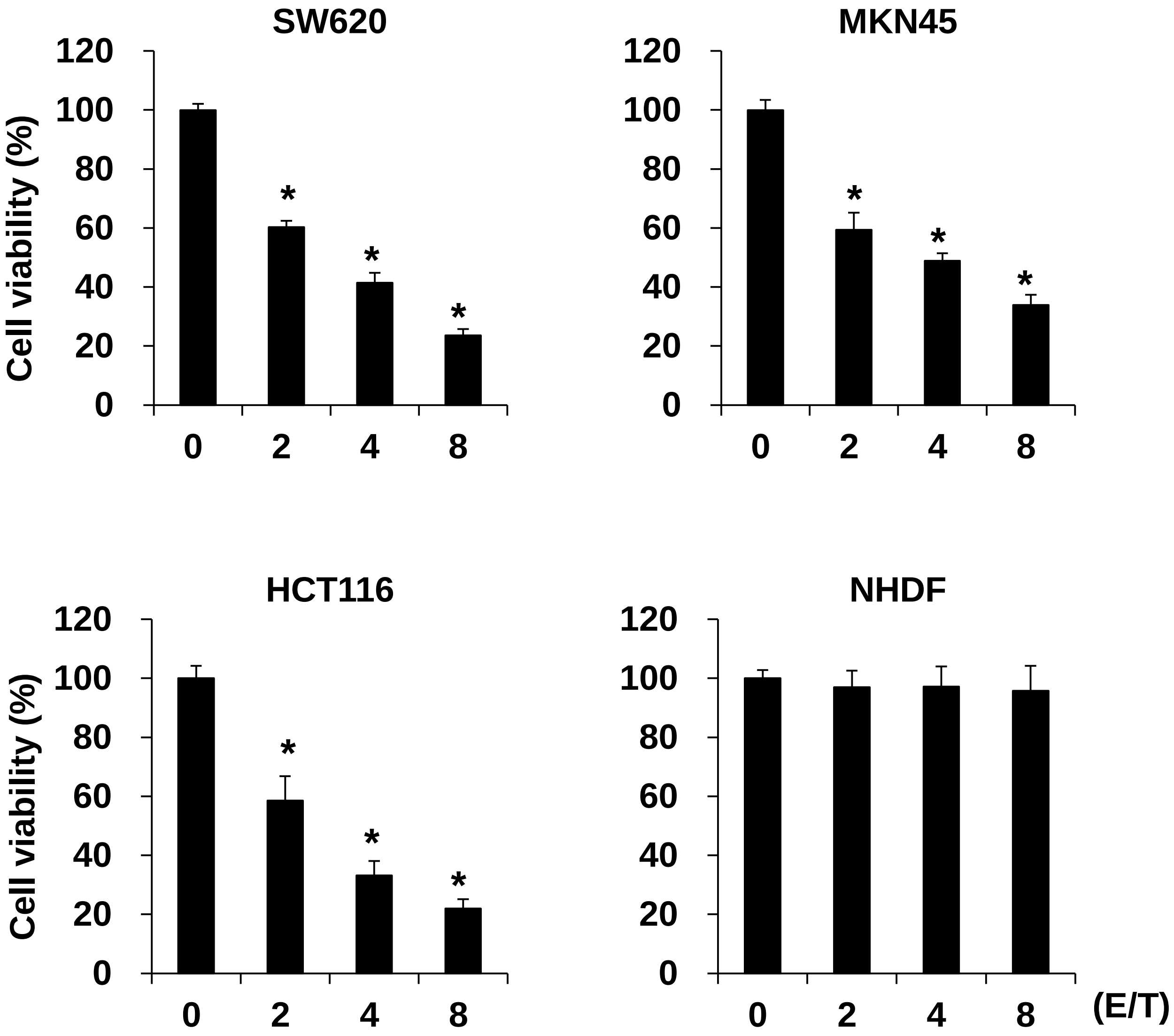

Supplementary Figure 3

HOZOT cell–mediated cytotoxic effect against human cancer cells and normal fibroblasts. Cells of 3 human cancer lines (SW620, MKN45, and HCT116) and NHDFs were treated with HOZOT cells at the indicated E/T ratio; cell viability was quantified using the XTT assay. Cell viability was calculated relative to that of the non-treated group, which was set at 100%. Cell viability data are expressed as mean values ± SD (n = 5). *, $P < 0.05$.

Supplementary Video 1

Time-lapse imaging spanning 48 hours after treatment showed that virus-loaded HOZOT-401/F35 cells induced GFP expression on the periphery of the tumor sphere composed of SW620 cells.

Supplementary Video 2

Time-lapse imaging spanning 5 days after treatment showed that HOZOT-401/F35 cells induced GFP expression and subsequent cell death in tumor spheres composed of SW620 cells.
